# Patients’ Perspectives on Dietary Patterns and Eating Behaviors During Weight Regain After Gastric Bypass Surgery

**DOI:** 10.1007/s11695-023-06718-9

**Published:** 2023-07-05

**Authors:** Liisa Tolvanen, Anne Christenson, Stephanie E. Bonn, Pamela J. Surkan, Ylva Trolle Lagerros

**Affiliations:** 1 Division of Clinical Epidemiology, Department of Medicine Solna, Karolinska Institutet, Maria Aspmans gata 30A, Stockholm, SE-171 64 Sweden; 2 Center for Obesity, Academic Specialist Center, Stockholm, Sweden; 3 Department of International Health, Johns Hopkins Bloomberg School of Public Health, Baltimore, MD USA

**Keywords:** Bariatric surgery, Eating habits, Nutrition, Support, Weight recurrence

## Abstract

**Purpose:**

Food quality, energy intake, and various eating-related problems have been highlighted as some of the components influencing weight after bariatric surgery. This study aimed to increase our knowledge of patients’ perspectives on dietary patterns and eating behaviors during weight regain after bariatric surgery.

**Materials and Methods:**

We recruited 4 men and 12 women with obesity and the experience of weight regain after bariatric surgery at an obesity clinic in Stockholm, Sweden. Data were collected during 2018–2019. We conducted a qualitative study, carried out individual semi-structured interviews, and analyzed the recorded and transcribed interview data with thematic analysis.

**Results:**

Participants had regained 12 to 71% from their lowest weight after gastric bypass surgery performed 3 to 15 years before. They perceived their dietary challenges as overwhelming and had not expected weight management, meal patterns, increasing portion sizes, and appealing energy-dense foods to be problematic after surgery. In addition, difficulties with disordered eating patterns, emotional eating, and increased alcohol intake further contributed to the weight management hurdles. Insufficient nutritional knowledge and lack of support limited participants’ ability to avoid weight regain, leading to restrictive eating and dieting without sustained weight loss.

**Conclusion:**

Eating behavior and dietary factors such as lack of nutritional knowledge, emotional eating, or disorganized meal patterns contribute to difficulties with weight management after gastric bypass surgery. Improved counseling may help patients prepare for possible weight regain and remaining challenges with food and eating. The results highlight the importance of regular medical nutrition therapy after gastric bypass surgery.

**Graphical Abstract:**

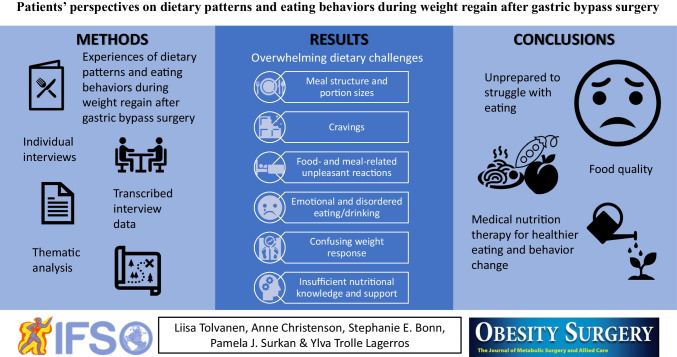

## Introduction

Bariatric surgery has been recognized as a safe, evidence-based treatment for obesity, contributing to long-term weight loss and improved health outcomes [1, 2]. However, some people experience a substantial weight gain from their lowest weight after surgery [3]. Even though the exact prevalence of weight regain still is uncertain due to the lack of a clear definition [4], some studies have suggested that approximately 20 to 24% of patients have regained 15% from their lowest weight five years after bariatric surgery [3, 5].

Factors influencing weight regain are complex and only partly explained [6]. Differences between individual weight outcomes may be partly genetic [7]. Furthermore, anatomical factors related to surgical failure, depression or other psychiatric conditions, disordered eating behaviors, and a sedentary lifestyle may contribute [6]. Physiological changes in the gut hormones affecting appetite regulation have been suggested to influence weight management [8]. In addition, the differences in response to the hedonic value of food may impact preferences in food intake [9]. Weight regain after bariatric surgery may also be related to how patients adjust their food intake and daily eating routines based on recommendations [10, 11].

Eating behaviors often improve after bariatric surgery. Patients may experience decreased appetite even 10 years after surgery [12], which contributes to smaller portions with lower energy density [13, 14]. In addition, patients appear more prone to eat fruits, vegetables, and fewer sugary and fatty foods [15], likely related to changes in taste and smell that favor healthier food choices [16]. All these factors may protect against weight regain. However, it may also be challenging to adopt food practices that facilitate sustained weight loss. Dietary patterns, including higher amounts of sweets, snacks, sugar-sweetened beverages, alcohol, and fatty foods, may promote weight regain [10, 11, 17]. In addition, disordered eating behaviors, such as picking, nibbling, and grazing, may complicate weight management and psychosocial well-being after bariatric surgery [18]. Furthermore, post-surgical emotional eating has also been shown to be associated with weight regain after bariatric surgery [19]. Stress eating is one example of a response to the dysregulation of negative emotions [20] that may affect weight management. Additionally, it has been suggested that people with poor post-surgical weight loss outcomes may perceive higher disinhibition and more hedonic hunger compared to those with sustained weight loss results [21].

In all, food quality, energy intake, and eating-related problems influence weight management after bariatric surgery. However, nutritional challenges during weight regain are complex and still poorly understood. There is a lack of qualitative studies exploring patients’ lived experiences of food and eating during weight regain post-surgically. Therefore, we aimed to further our understanding of patients’ perspectives on dietary patterns and eating behaviors during weight regain after bariatric surgery.

## Materials

We purposively sampled participants from a specialty obesity clinic in Stockholm, Sweden, between 2018 and 2019. Eligible participants had previously undergone bariatric surgery (gastric bypass or sleeve gastrectomy) and regained at least 10% from their lowest post-surgical weight. They were ≥ 18 years of age, had a body mass index (BMI) ≥ 35 kg/m^2^, and were Swedish speaking. Patients received oral and written study information and signed written informed consent. Participation was voluntary and did not influence patients’ clinical treatment. Participants did not receive any incentive. Three female patients declined participation, because of time constraints. They were somewhat younger (mean 37 years vs. 49 years), but had similar BMI and amount of regained weight, compared to the participants included.

## Methods

We explored participants’ perceptions and experiences of weight regain after bariatric surgery through in-depth semi-structured individual interviews. The interview guide contained background questions about participants’ age, type and date of bariatric surgery, weight change after surgery, health status, and family situation. In addition, the interview guide covered open-ended questions related to experiences of weight regain after bariatric surgery and support during weight regain. We also asked complementary questions to stimulate participants’ narratives and to gather additional information about their experiences and perceptions. We have previously reported on general experiences of weight regain [22] and perceptions of social support [23] from this data set. However, participants’ narratives included extensive descriptions of dietary and eating-related challenges. These subjective experiences served as the basis for the analysis in the present study.

All interviews took place at the obesity clinic and were conducted in Swedish by the first author (LT) and lasted, on average, 60 min (range 32–79 min). The first author transcribed the recorded interviews verbatim. We strived for a variation in age, sex, and country of origin among participants. We assessed that 16 interviews were enough to reach informational redundancy [24].

### Data Analysis

We analyzed the written interview data with thematic analysis by Braun and Clarke [25, 26] to capture participants’ voices and their realities. In the thematic analysis, textual data were systematically coded and grouped while formulating sub-themes and themes, capturing the essence of participant narratives. We strived to be reflexive about our preconceptions and assumptions of the phenomenon studied. All authors are females with research experience in the field of obesity, and the first (LT), second (AC), and last authors (YTL) are also healthcare professionals who provide specialty obesity care. The first and the second author collaborated and discussed suggestions for initial codes and themes to enhance reflexivity and interpretation. Finally, all co-authors reviewed the themes and sub-themes and added their perspectives to the process.

## Results

### Participants

Participant characteristics are presented in Table 1. In total, 4 men and 12 women were included, with a mean age of 49 years and an average weight regain of 36% from lowest weight after gastric bypass surgery. They undertook the operation between 2004 and 2016. Three participants underwent sleeve gastrectomy (*n* = 1) or laparoscopic gastric banding (*n* = 2) prior to their gastric bypass surgery. Most participants (*n* = 13) self-reported noticing weight regain starting between 1 and 5 years (on average 2.6 years) after gastric bypass surgery; others (*n* = 3) could not recall when their weight regain began.

**Table 1:** Participant characteristics (*n* = 16)

	Mean	(Range)
Age, years	49.0	(20–64)
Body mass index, BMI kg/m^2^		
Pre-gastric bypass^a^	52.0	(42–70)
At nadir	34.0	(25–49)
At the interview	46.0	(36–66)
Time since gastric bypass, years	10	(3–15)
	%	(Range)
Weight change		
Percentage of total weight loss from gastric bypass (%TWL)	35.0	(14–50)
Percentage of total weight recurrence from nadir (%TWR)	36.0	(12–71)
Percentage of excess weight loss (%EWL)		
At nadir	70.0	(28–98)
At the interview	25.0	(− 20–54)
	*n*	(%)
Sex		
Women	12	(75.0)
Men	4	(25.0)
Country of birth		
Nordic countries	11	(69.0)
Outside of Europe	5	(31.0)
Employment		
Employed/studied	13	(81.0
Other income, e.g., income support/sick leave	3	(19.0)
Civil status		
Married/in a relationship	12	(75.0)
Single	4	(25.0)

^a^Gastric bypass was a second bariatric surgery for three participants (previous surgeries: sleeve gastrectomy *n* = 1, gastric banding *n* = 2)

### Theme and Sub-themes

We generated one main theme, *overwhelming dietary challenges*, and six sub-themes during the thematic analysis (Table 2). The mark “/…/” indicates that unrelated text has been removed within quotes presented.

**Table 2:** Summary of the results; theme, sub-themes, and example of codes

Theme	Sub-themes	Example of codes
Overwhelming dietary challenges	Meal structure and portion sizes	Less structure and fewer meals; increased hunger during pregnancy at nighttime; night sandwiches when depressed; increased hunger and larger portions; big differences in portion sizes; still small portions; forgetting to eat without a reminder; eating nothing when stressed; being able to eat more contributes to the feeling of failure
	Cravings	Testing limits; prefers to eat sweets; over time more chips and sweets; constant cravings, escalation of unhealthy eating behaviors; sugar-sweetened beverages; increased intake of sugar; able to eat ice cream and sweets; cravings for chocolate during pregnancy; the desire for food was unchanged; grazing carbohydrates; could not stop cravings and reduce the fat intake
	Food- and meal-related unpleasant reactions	Diarrhea and nausea; vomiting of healthy food; nausea from steak; tiredness after a meal; dumping when eating out with friends; takes dumping and ignores it; grazing to minimize dumping; difficult reaction (dumping) when overeating; reduced quality of life because not being able to eat well; food brings no pleasure
	Emotional and disordered eating/drinking	Comfort eating to manage negative thoughts; difficult to control eating; comfort eating when feeling lonely; anxiety drives eating; alcohol to cope with emotions, alcohol decreases emotional pain; self-harm behavior by eating too little; the eating disorder remains after surgery; did not change eating behavior; punishing myself with secret eating; unhealthy relationship with food
	Confusing weight response	My body must be starving; something must be wrong with my body; eats like a bird; avoids sweets, candies, and chocolate; healthcare professionals do not believe me; eating nothing and still not losing weight; no metabolism; frustrated that the weight is stable despite of healthy habits; dietitian thinks I eat too little; trying to eat healthily
	Insufficient nutritional knowledge and support	Lack of knowledge about nutrition; lack of information on energy content; missing information about the risk of weight gain; difficult in understanding nutrition education; need for concrete advice on portion sizes and food choices; misinformed about what to eat after surgery; need for trustworthy advice on eating behaviors and weight management; insufficient information

#### Overwhelming Dietary Challenges

The main theme illustrates a profound lack of capacity to meet post-surgical dietary challenges. Specifically, participants were unprepared for weight regain and to discover that eating- and drinking-related difficulties would remain. They trusted that the effect of the surgery would lead to significant and sustained weight loss and found it frustrating and disappointing when new obstacles appeared, such as altered effects of alcohol and food- and meal-related problems. Participants criticized and blamed themselves for having difficulties maintaining healthy eating habits.

### Sub-themes

#### Meal Structure and Portion Sizes

Participants had a general knowledge of the importance of regular meal patterns. However, most of them changed from more frequent to fewer meals over time. For example, some only ate main meals (e.g., breakfast, lunch, and dinner), others skipped breakfast or snacks, and some practiced grazing throughout the day.

I graze again. And I am eating the wrong things when I graze. (Participant 5)

The frequency and duration of meals was challenging in times of stress. Parents with young children emphasized that it was difficult to prepare and eat meals when experiencing stress in family life. Furthermore, some participants lost their appetite, had trouble swallowing food, or ate nothing when stressed.

Because if I’m stressed and…well, one is stressed and will eat, and we should hurry to eat lunch quickly. Then I can skip eating. Because as soon as I’m stressed, the body feels it, and then it blocks up. It feels like a blockage in the stomach. (Participant 7)

In addition, their families’ eating habits affected participant meal structure and dietary patterns.

…that they [the family] shouldn’t have to eat the same thing that I eat and stuff like that, /…/ I knew about timing and that you must keep a meal structure, but I ate the same food as them. (Participant 1)

Some participants described eating at night. Working hours also affected meal patterns.

…it [shift work] slightly disturbs the balance, especially if you have worked in the evening and must get up early the next day. Then you’re tired, and you don’t feel like eating breakfast. (Participant 14)

Initially, after gastric bypass surgery, participants experienced a restriction in eating that led to reduced portion sizes. However, portion sizes gradually increased as the feeling of restraint diminished. Different participants described various portion sizes; some ate extremely little, and others described increased hunger and large portions, although surgery still helped most of the participants to eat smaller portions.

And I can eat more now. I can do that. So, my stomach has gotten bigger. I can’t eat a full normal portion, but half, maybe 75 percent even. (Participant 9)

#### Cravings

Contrary to their expectations, cravings did not disappear after surgery. Instead, some could eat fatty and sweet foods almost immediately post-surgically.

I had hoped that the surgery would put a stop to my sweet tooth, that I would be unable to eat sweets and fat and able to stick to small meals. But that quickly became a letdown. (Participant 5)

Participants who experienced fewer cravings after surgery were curious to test if eating sweets would cause unpleasant side effects.

So, the way you learn where your limits are, you’re like a kid again and testing the limits. (Participant 15)

Pregnancy, mental health problems, and social eating were perceived as particularly challenging leading to consumption of high-energy-dense foods and drinks. Increased intake of sweets and fatty foods induced feelings of anxiety and guilt and promises to oneself to exert more “willpower” the next day.

Then you start feeling bad, but since you already have eaten badly, you might as well continue. So, during the day, especially in the evenings, you think, ‘Tomorrow, tomorrow I’m not going to eat, then I’m going to eat really healthy and well’. Then you get up in the morning and ‘Ah, I need something quickly’, and stuff it in. (Participant 8)

#### Food- and Meal-Related Unpleasant Reactions

Challenges with food- and meal-related gastrointestinal reactions such as constipation, diarrhea, and nausea were burdensome. Participants highlighted foods rich in fat, sugar, and meat as particularly problematic.

I feel sick as soon as I eat a little, for example, cheese snacks or stuff myself with chips or something like that. Immediately, I feel sick, and I get diarrhea at once. (Participant 4)

Some participants experienced, as an only symptom, inexplicable fatigue that required bed rest after meals. They compared this with the symptoms in dumping syndrome (a reaction that may include feeling nauseous, tired, or dizzy when undigested food rapidly passes from the stomach pouch to the small intestine) and stated that this fatigue was different. Nevertheless, dumping was also a problem for participants, and they used strategies such as grazing, eating slowly, resting, or avoiding foods that caused discomfort. For some participants, dumping and early satiety were problematic phenomena in social situations as they caused worry about eating in front of others. These reactions could, in turn, harm participants’ enjoyment of and relationship with food.

You want to enjoy food; I never enjoy any food. I feel sick when I look at food. I just, I hate food. (Participant 12)

To some extent, dumping syndrome could limit the intake of sweets. However, for several participants, cravings made them eat sweets regardless of dumping. In addition, the intensity of dumping diminished with time, and they learned what they could eat to avoid dumping.

And I knew I would get dumping, but I ate anyway. I had a hard time resisting because the craving was stronger. (Participant 6)

#### Emotional and Disordered Eating/Drinking

Several participants had current or previous mental health problems and used food or alcohol for emotional relief. Loneliness, anxiety, and depression triggered emotional eating and participants lacked alternative strategies for dealing with negative emotions.

It’s just that this feeling of worry maybe, some kind of anxiety. The anxiety inside, unconsciously controlling me. That I constantly, that I regularly must have something [to eat]. (Participant 3)

Family members would sometimes comfort them with sweets or, on the contrary, make negative comments about their eating behavior that could lead to secret eating of “forbidden” foods. For some participants, their emotional pain could be so intense that they preferred the negative consequences of dumping in favor of the comforting effects of food.

If you want you can learn to handle this [dumping] if you feel bad enough. /…/ I have at times eaten a lot of sweets, felt bad [got dumping] but did it anyway because I have felt so bad [mentally/emotionally] that it has been my way to cope with it. (Participant 15)

Some participants described disordered eating behaviors, and some defined themselves as food or sugar addicted. Meanwhile, healthcare professionals did not consistently detect psychological problems with eating during the follow-up visits.

I [during the follow-up visit at the surgical clinic] was caught up with feeling ashamed about having to learn how to eat right. That was the main thing. So, I got no psychological [assessment/treatment]. (Participant 3)

Additionally, participants had been reluctant to reveal problematic eating behaviors during the pre-surgical assessment, partly due to fear that it would stop them from getting the surgery.

Of course, they [healthcare professionals] ask, ‘you shouldn’t have any eating disorders’, but who says ‘of course, I have an eating disorder’ if you get a chance to get surgery and be able to get better. (Participant 8)

Some participants increased their alcohol consumption post-surgically. Social drinking was perceived as a way to obtain social status, especially in young adulthood, but also to deal with negative emotions.

The alcohol relieves the pain. Yes. /…/ Initially, it was for partying, and then it [drinking] became the point itself. (Participant 16)

#### Confusing Weight Response

Participants had anticipated losing weight permanently and were unprepared to regain weight. Hence, it was a disappointment when weight regain started.

Increasing weight induced dieting plans and restrictive eating behaviors. However, despite attempts to limit energy intake and be physically active, they were frustrated when no weight was lost, or it felt impossible to keep off.

I don’t eat anything, anyway… then I think now I’m going to lose weight. But no, I’m not losing weight. It’s weird. Feels like there is something wrong with me. (Participant 12)

Since participants perceived weight loss as almost impossible after weight regain, they questioned whether this could be due to possible physical problems such as slow metabolism as a response to starvation, aggravating genetic factors, or surgery-related anatomical changes. Healthcare professionals were perceived as ignorant in treating weight regain.

She [the dietitian] was just as confused and agreed that I should be losing weight [based on to my food intake]. (Participant 8)

Participants felt that healthcare professionals met them with disbelief when they reported low energy intake. Feelings of being discredited were also described when eating with friends and when eating less than everyone else, as a participant stated she ate “like a bird.”

#### Insufficient Nutritional Knowledge and Support

Participants expressed varied awareness of how they should eat to manage their weight post-surgically. Some seemed to have insufficient knowledge regarding dietary patterns such as portion sizes, meal structure, the altered effects of alcohol, or macronutrients that are important after gastric bypass surgery.

It might sound a bit stupid, but I don’t know about calories, protein, or what they are or anything like that. So, when they talked about carbs, I understood zero. Because I didn’t know what it was. And I wanted that information to understand what they were talking about. (Participant 6)

Participants requested a thorough assessment of their eating behaviors as well as trustworthy, individualized nutritional strategies to prevent or treat weight regain. Some participants felt misinformed about the possibility of weight regain, and that energy intake could increase over time.

At the time I did not consider that there is still a lot of sugar and stuff [in sweets], so I didn’t realize [that the body would adapt to increased intake of sugar]. Instead, I believed that now I would be getting a smaller stomach, and I would never again be able to eat this much. (Participant 13)

Some participants had not taken vitamin and mineral supplements for several years, causing additional challenges with deficiencies and related conditions such as osteoporosis.

According to the participants, the pre-surgical information, delivered mainly as group information, only focused on the surgical procedure and dietary recommendations in conjunction with the surgery. All but one had received some follow-up visits by nurses or dietitians during the first 2 years after the surgery, indicating some interdisciplinary follow-up. Post-surgical follow-ups in primary care were described as scarce and unsatisfactory. Some participants were offered follow-ups 5 and/or 10 years post-surgically at the surgical clinic. However, psychological support was perceived as non-existent and nutritional support insufficient, contributing to dietary challenges.

Maybe you [referring to herself] should also meet a counselor or psychologist because you still have a remaining [problematic] eating behavior like mine. I couldn’t eat any proper meals because I got dumping, I took a small piece of bread or something all the time, little things. So, more control [nutritional- and psychological support], not just, ‘Manage this on your own’. (Participant 8)

One participant expressed being satisfied with support from interdisciplinary healthcare professionals both pre- and post-surgically. Participant appreciated that the surgeon examined her stomach pouch after weight regain.

It [follow-up] has been good. They [healthcare professionals] gave good information. They make appointments at certain intervals to check if everything is working well. /…/ It was great. I was satisfied. /…/ They gave me all the examinations, x-rays, and everything. And he [the surgeon] said: ‘Everything looks really good, nothing strange, ‘the stomach’ is as we made it’. (Participant 10)

Self-blame for weight regain and dietary challenges were prominent in participants regardless of the extent of support they had received. Nevertheless, most participants perceived contact with healthcare professionals as insufficient, not satisfying their need for knowledge or skills to overcome problems related to eating behaviors.

It was [nurse’s name] who was in charge of all these patients [undergoing bariatric surgery], but she was not a good person. She was completely uninterested. She didn’t give advice, food lists, restrictions, or anything else that patients get nowadays. (Participant 12)

## Discussion

In the present qualitative study, participants with obesity and weight regain after gastric bypass surgery expressed *overwhelming dietary challenges*. They described overall unpreparedness and disappointment that eating- and drinking-related problems remained. Participants had not expected to regain their lost weight after surgery. They expressed insufficient nutritional knowledge and lacked support and adequate behavioral tools to resist weight regain. Participants blamed themselves for the difficulties with dietary patterns and eating behaviors.

Unrealistic weight loss expectations are common in patients undergoing bariatric surgery [27, 28]. Studies have reported that patients expect to lose nearly all (90–106%) of their excess body weight (percent excess weight loss, %EWL) [27, 29]. Women in Sweden have been shown to have higher expectations of post-surgery weight loss compared to women in other European countries [30]. Furthermore, women and patients with higher BMIs expect to lose a substantial amount of weight [27], which aligns with the participant characteristics in the present study, where several of the participants also expressed that they were dissatisfied with the result. Unrealistic expectations may contribute to feelings of shame among patients with weight regain [31], and lead to problematic eating behaviors as a reaction to difficulties in maintaining weight [32].

For the majority of patients, bariatric surgery leads to sustained weight loss [33]. Though weight loss outcomes are individual, meta-analyses have shown long-term results of 68% EWL at 2 years follow-up [34] and 57% EWL at 10 years post-gastric bypass surgery [33]. Our study participants reported similar levels of weight loss, with an average excess weight loss of 70%, ranging from 28 to 98% EWL, at nadir.

Most patients achieve their lowest weight at approximately 12 to 18 months post-surgically and have often stabilized their weight 2 years after surgery [35]. Some weight regain is expected [3], and a small proportion of patients begin regaining weight as early as 6 months after gastric bypass surgery [35]. Participants in the present study started to regain weight on average 2.6 years after their surgery. This is in line with previous research showing that 2 years post-surgery appears to be a sensitive period for weight regain to start [36]. This tends to be at the same time when most patients are referred to primary care for long-term follow-up in accordance with guidelines [37].

The Swedish Obesity Surgery Registry reported a drop in follow-up attendance from 95% at 6 weeks to 63% at 2 years [38]. A systematic review and meta-analysis reported a significant association between follow-up attendance and %EWL 3 years post-surgery [39]. Attrition from yearly follow-up may be due to different reasons, such as work- or family-related issues [40]. Difficulties in weight management and weight regain may also contribute to higher attrition [40, 41]. However, most participants in the present study desired more support during weight regain, especially nutritional and psychological support.

Some participants in our study reported that they could eat energy-dense foods almost immediately after surgery without problems such as dumping. Early dumping may control eating behavior [42], and thereby facilitate weight maintenance. Results from the present study indicate that cravings and emotional pain may sometimes be so intense that participants give in and expose themselves to sweets, savory, and fatty foods, with subsequent dumping, contributing to inadequate food quality, and weight regain. In summary, our study confirms the value of detecting underlying factors that affect food choices, meal patterns, and portion sizes.

Participants shared their experiences of emotional and disordered eating and drinking behaviors. These narratives align with previous studies showing that problematic eating behaviors may contribute to weight regain [43–45]. However, binge eating behaviors are often reduced due to anatomic restrictions after bariatric surgery. Instead, it has been suggested that patients might have difficulties with loss-of-control eating or subjective binge eating behavior [46]. In subjective binge eating, patients perceive that they are consuming a large amount of food, while in fact, they only eat a little or moderate amount of food. Weight regain has also been associated with eating behaviors such as grazing, picking, and nibbling [45, 46]. The present study indicated that patients might be reluctant to discuss problematic eating behaviors with healthcare professionals before surgery. On the other hand, they indicated a lack of psychological assessment and treatment during the follow-up visits. These results suggest that healthcare providers should raise patients’ awareness of potentially problematic pre- and post-surgical behaviors and offer adequate treatment when needed.

Participants experienced insufficient nutritional knowledge while concurrently perceiving difficulties with accessing medical nutrition therapy (MNT). They returned to dieting and restrictive eating strategies to control their weight and lacked adequate nutritional support. The lack of nutritional counseling and low compliance to follow-ups with healthcare professionals after bariatric surgery is concerning and associated with weight regain [10, 11].

Participants self-reported information about their contacts with healthcare services, but we did not have access to their pre- or postoperative medical charts to verify the information. It is possible that they might have forgotten details regarding the frequency or content of the follow-up visits, considering that the surgery was conducted 3–15 years before the interview. This may be seen as a limitation. Nordic and other international guidelines and recommendations have stated the importance of life-long regular follow-up, monitoring, and access to an interdisciplinary team after bariatric surgery [37, 47–49]. In these guidelines, frequent contact with a registered dietitian is highlighted to improve nutritional outcomes among bariatric surgery patients. However, participants in the present study reported occasional or absent contact with a dietitian.

*The Nordic guidelines for monitoring and supplementing with vitamins/minerals and follow-up after obesity surgery* [37] were published in 2017, i.e., after all the participants in the present study had undergone surgery. The absence of guidelines may partly explain differences in nutritional follow-up and the insufficient nutritional knowledge that was perceived by the participants. The Nordic guidelines recommend follow-ups at 6 weeks, 3 and 6 months, and 1- and 2-year visits at surgical clinics [37]. Long-term annual follow-ups (> 2 years) are recommended to be conducted within primary care by dietitians, physicians, and/or nurses with good knowledge of bariatric surgery. Additional routine visits at the surgical clinic are recommended at years five and ten post-surgically. Follow-up includes assessing possible complications and co-morbidities, as well as providing nutritional monitoring to improve long-term outcomes.

It has been suggested that registered dietitians should provide medical nutrition therapy at least every 3 months during the first year, twice during the second year, and yearly in the long term to improve nutritional outcomes, weight loss, and healthier eating behaviors [50]. This type of follow-up may be helpful when patients struggle with different nutrition-related problems, including weight regain. Patients that regain weight may benefit from additional treatment, such as psychological interventions [51], anti-obesity medications [52], or additional surgical options [53].

A strength of our study is that participants had first-hand experiences of weight regain after gastric bypass surgery, which increases the study’s trustworthiness and credibility [24]. The study population varied in age, sex, and origin, which could increase our results’ transferability to other groups. Twelve participants (corresponding to 75% of the total) were women, which is slightly less than the proportion of women compared to men that undergo bariatric surgery in Sweden [54]. The high BMI (mean 46 kg/m^2^ after weight regain) of our participants may limit the transferability of our results to a lower BMI range. Still, we included participants with a wide range of BMI from 36 to 66 kg/m^2^.

We explored perceptions only after gastric bypass surgery, excluding other similar procedures. Experiences of dietary patterns and eating behaviors may differ after other types of bariatric procedures. In Sweden, gastric bypass surgery has been the dominant procedure, but recent reports show that gastric bypass and sleeve gastrectomy were nearly equally performed (48.8% vs. 45.7%) in 2021 [54]. Gastric bypass has shown to be the surgical method with long-lasting weight loss maintenance.

## Conclusions

Some patients with weight regain were exposed to overwhelming dietary challenges following gastric bypass surgery. They were unprepared for the struggle with eating habits, cravings, and emotional and disordered eating behaviors. Furthermore, they lacked sufficient nutritional knowledge, support, and adequate behavioral tools to resist weight regain. There was wide variation in the timing of when dietary challenges appeared postoperatively. Our results highlight the importance of an interdisciplinary approach including medical nutrition therapy and psychological support to assess and monitor patients so that they can achieve healthier eating patterns and necessary behavioral changes after gastric bypass surgery.

## Call to Action

Various dietary- and eating behavior-related difficulties may challenge patients with weight regain after gastric bypass surgery. The education of healthcare professionals, especially primary care physicians and dietitians, in pre- and post-surgical assessment and follow-up should be included in clinical guidelines. Registered dietitians should provide medical nutrition therapy pre- and post-surgically to target patients’ expectations, long-term weight loss outcomes, and nutritional challenges. Pre- and postoperative protocols may need adjustments to be customized and people-centered for assessment, monitoring, and long-term follow-up. An interdisciplinary approach may tailor underlying multifactorial causes of weight regain. This qualitative study has generated several hypotheses that could be tested in future studies with the aim to develop behavioral interventions and best practices in medical nutrition therapy to prevent and treat weight regain after gastric bypass surgery.
